# Metabolic Profiling of the Hypothalamus of Mice during Short-Term Food Deprivation

**DOI:** 10.3390/metabo12050407

**Published:** 2022-04-29

**Authors:** Ye Jin Kim, Dasol Kang, Hye Rim Yang, Byong Seo Park, Thai Hien Tu, Bora Jeong, Byung Ju Lee, Jae Kwang Kim, Jae Geun Kim

**Affiliations:** 1 Division of Life Sciences, College of Life Sciences and Bioengineering, Incheon National University, Incheon 22012, Korea; 201721047@inu.ac.kr (Y.J.K.); hr.yang0414@inu.ac.kr (H.R.Y.); 2021s135@inu.ac.kr (B.S.P.); thaihientu@gmail.com (T.H.T.); 2 Department of Biological Science, University of Ulsan, Ulsan 44610, Korea; lainef7@nate.com (D.K.); boraring@naver.com (B.J.); bjlee@ulsan.ac.kr (B.J.L.)

**Keywords:** food deprivation, hypothalamus, metabolites, astrocyte, monocarboxylate

## Abstract

Nutrient availability and utilization in hypothalamic cells are directly associated with the regulation of whole-body energy homeostasis. Thus, establishing metabolic profiling in the hypothalamus in response to metabolic shift is valuable to better understand the underlying mechanism of appetite regulation. In the present study, we evaluate the alteration of lipophilic and hydrophilic metabolites in both the hypothalamus and serum of fasted mice. Fasted mice displayed an elevated ketone body and decreased lactate levels in the hypothalamus. In support of the metabolite data, we further confirmed that short-term food deprivation resulted in the altered expression of genes involved in cellular metabolic processes, including the shuttling of fuel sources and the production of monocarboxylates in hypothalamic astrocytes. Overall, the current study provides useful information to close the gap in our understanding of the molecular and cellular mechanisms underlying hypothalamic control of whole-body energy metabolism.

## 1. Introduction

The maintenance of energy metabolism in brain cells is essential for normal brain function [1]. Thus, impaired metabolic activity can be used as a pathological sign for understanding the initiation and development of neurological disorders [2]. The hypothalamus is the central unit for controlling energy balance by sensing whole-body energy levels and operating various metabolically active organs [3]. Metabolic alterations in hypothalamic cells drive the behavioral phenotypes linked to the control of whole-body energy metabolism [4,5,6,7]. Furthermore, altered metabolic processes, such as dynamic changes in mitochondrial activity and utilization of carbohydrates and lipids, are observed in hypothalamic cells of rodent models of obesity [3,4,7,8]. In line with this evidence, several research groups have reported that altered shuttling and biosynthesis of fuel sources in hypothalamic glial cells led to changes in metabolic controls, thereby exacerbating obesity pathogenesis [5,6]. However, the profiling of metabolites in the hypothalamus in response to metabolic shifts has not yet been investigated.

Astrocytes, which are the most abundant cell type in the central nervous system, primarily contribute to supporting normal brain functions by buffering neurotransmitters and supplying energy sources [1,9]. Intriguingly, hypothalamic astrocytes primarily coordinate energy metabolism by regulating synaptic input organization onto target neurons, such as agouti-related peptide (AgRP) and proopiomelanocortin (POMC) [6,10,11]. In the present study, we investigate the levels of lipophilic and hydrophilic metabolites in both the serum and hypothalami of fasted mice to determine the nutritional factors involved in appetite regulation using gas chromatography-mass spectrometry (GC-MS). We further determine the astrocyte-specific expression of genes involved in cellular metabolic processes to confirm the metabolomics data using a Ribo-Tag method. Overall, the current study provides molecular and biochemical information to better understand the dynamics of metabolic interactions between hypothalamic neurons and glial cells that govern whole-body energy homeostasis.

## 2. Results

### 2.1. Altered Fatty Acid and Hydrophilic Metabolite Levels Were Observed in the Serum of Fasted Mice

**Figure 1:**
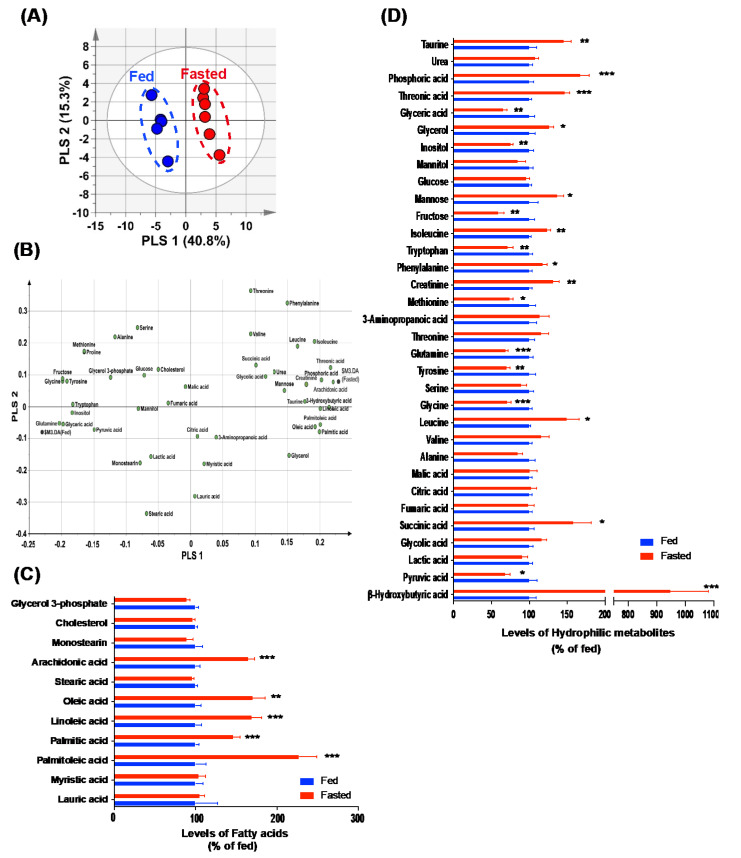
Metabolic profiling in the serum of fasted mice. Levels of serum metabolites were measured in control and fasted mice using GC−MS. (**A**) Score and (**B**) loading plots of the PLS−DA model were obtained from 44 metabolites. (**C**) Most fatty acids displayed higher levels in the serum of fasted mice, compared to those in the serum of control mice. (**D**) A normalized graph showing altered hydrophilic metabolites in the serum of fasted mice, compared to those in the control mice. Data are presented as the mean ± SEM. *n* = 5 mice in the control group and *n* = 6 in the fasted group. * *p* < 0.05, ** *p* < 0.01, *** *p* < 0.001.

For the serum metabolite profiling, we evaluated the levels of lipophilic and hydrophilic metabolites in the serum extracted from fasted and control mice by performing GC−MS analysis, and determined the overall pattern in complex experimental data by partial least square discriminant analysis (PLS−DA). The score for the first PLS component (PLS 1), which accounted for the largest possible variance in the dataset, was 40.8%. The second component (PLS 2) accounted for 15.3%. The score plot showed a clear separation for PLS 1 between the two groups, indicating a distinct pattern of serum metabolites (Figure 1A). In addition, the value of the variable importance in projection (VIP) in the PLS−DA model identifies metabolites that make important contributions to the differences between groups. Twenty-four serum metabolites with VIP values >1.0 were identified as significant contributors between fasted and control mice (Figure S1A). Among the serum metabolites detected, nine metabolites, namely, arachidonic acid, threonic acid, β-hydroxybutyric acid, phosphoric acid, palmitoleic acid, linoleic acid, palmitic acid, oleic acid, and isoleucine, were the major positive PLS 1-loading metabolites, indicating that their levels were higher in fasted mice. In contrast, seven metabolites, namely, glutamine, fructose, glycine, glyceric acid, tyrosine, inositol, and tryptophan, were negative PLS 1-loading metabolites, indicating that their levels in the serum of fasted mice were lower than in those in the serum of control mice (Figure 1B). In the normalized graphs, the levels of several fatty acids were elevated in the serum of fasted mice (Figure 1C), compared to those in the serum of fed control mice, while hydrophilic metabolites showed a bidirectional pattern of normalized data (Figure 1D). Notably, we observed a drastic elevation of β-hydroxybutyric acid, a ketone body, in the serum of fasted mice (Figure 1D).

### 2.2. Changes in the Hydrophilic Metabolites Were Found in the Hypothalami of Fasted Mice

**Figure 2:**
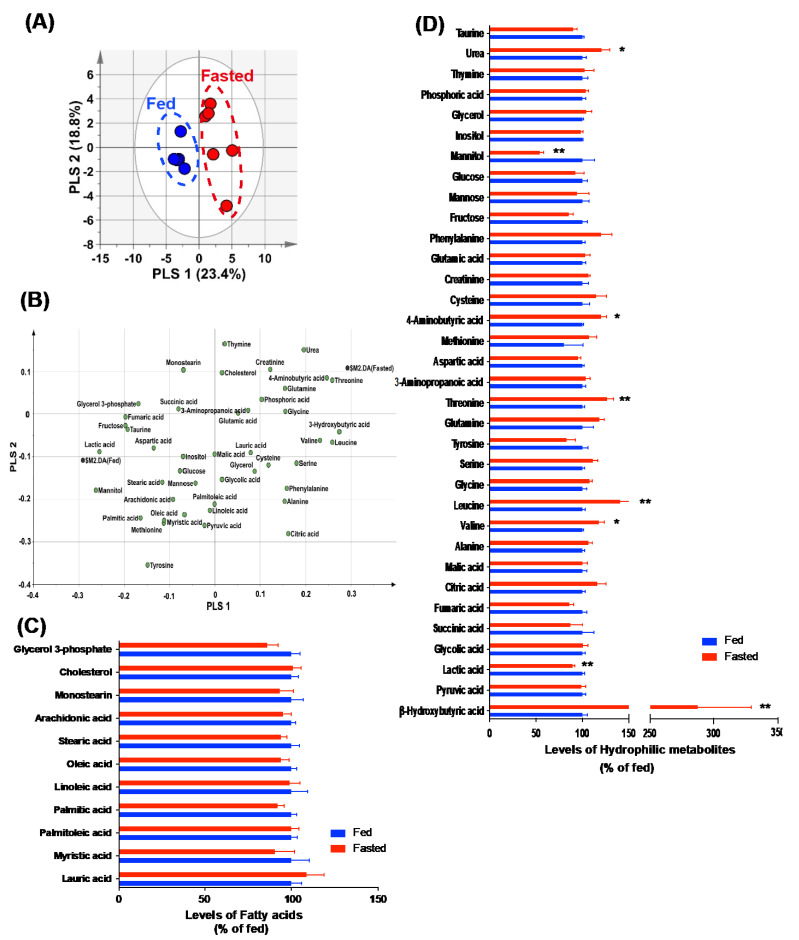
Metabolic profiling of the hypothalami of fasted mice. Hypothalamic metabolites were analyzed in the control and fasted mice using GC−MS. (**A**) Score and (**B**) loading plots of the PLS−DA model were obtained from 45 metabolites. (**C**) No difference in the levels of hypothalamic fatty acids was observed between the fasted and control mice. (**D**) A normalized graph showing altered hydrophilic metabolites in the hypothalami of fasted mice, compared to those in control mice. Data are presented as the mean ± SEM. *n* = 5 mice in the fed group and *n* = 6 in the fasted group. * *p* < 0.05, ** *p* < 0.01.

GC-MS analysis was performed on samples of the whole hypothalamus from control and fasted mice to establish the profiling of hypothalamic lipophilic and hydrophilic metabolites in response to short-term food deprivation. In the PLS−DA model, PLS 1 and 2 of the score plot explained 42.2% of the total variance (PLS 1, 23.4%; PLS 2, 18.8%). In the two-dimensional score plot, the *x* axis (PLS 1) separated the fasted hypothalami from the control hypothalami (Figure 2A). In addition, eight metabolites with VIP values > 1.0 and *p* < 0.05 were identified (Figure S1B). The positive PLS 1-loading metabolites in the separation between the two groups were β-hydroxybutyric acid, threonine, leucine, 4-aminobutyric acid, and valine. The negative PLS 1-loading metabolites were lactic acid and mannitol (Figure 2B). Inconsistent with the serum data, no significant difference in hypothalamic fatty acids was found between the control and fasted mice (Figure 2C). In addition, the majority of altered hydrophilic metabolites tended to be higher in the fasted hypothalami than in the control hypothalami (Figure 2D). Intriguingly, short-term food deprivation resulted in a dramatic increase in β-hydroxybutyric acid levels and a significant decrease in lactate levels in the hypothalami (Figure 2D). The results of Pearson’s correlation analysis were visualized using hierarchical clustering analysis to evaluate the correlation between the forty-five metabolites identified in the hypothalamus in the two groups. As shown in Figure S2, the correlation between β-hydroxybutyric acid and lactate was negative (r = −0.4752, *p* < 0.05). These results suggest that the opposite pattern of monocarboxylate content in the hypothalami could be nutritional factors that affect the hypothalamic control of energy metabolism.

### 2.3. Expression Levels of the Hypothalamic Genes Involved in the Metabolic Processes Respond to the Short-Term Food Deprivation

**Figure 3:**
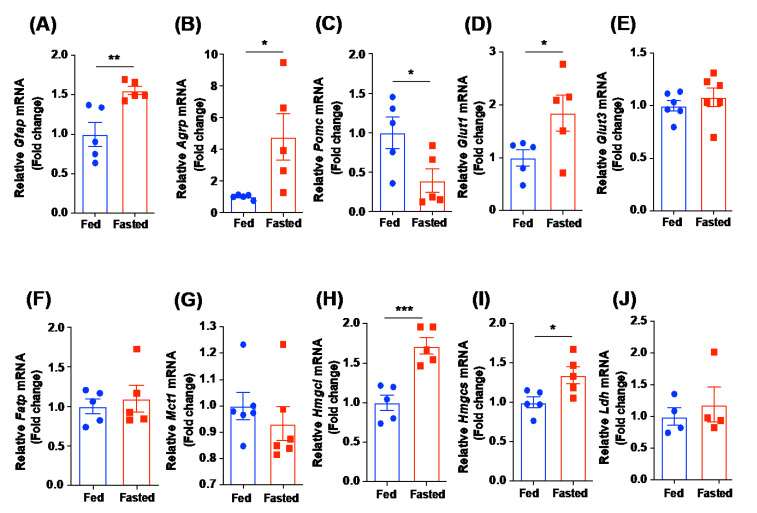
Altered mRNA levels of genes involved in metabolic processes in the hypothalamus in response to short-term food deprivation. qPCR experiments were performed with the hypothalami isolated from control and fasted (overnight) mice. Fasted mice showed an increase in hypothalamic mRNA levels of (**A**) *Gfap* and (**B**) *Agrp* and a decrease in hypothalamic mRNA levels of (**C**) *Pomc*. No significant alteration was found in the hypothalamic mRNA levels of (**E**) *Glut3*, (**F**) *Fatp*, (**G**) *Mct1*, and (**J**) *Ldh*. Fasted mice showed a significant increase in mRNA levels of (**D**) *Glut1*, (**H**) *Hmgcl*, and (**I**) *Hmgcs*. Data are presented as the mean ± SEM. *n* = 4–6 mice per group. * *p* < 0.05, ** *p* < 0.01, *** *p* < 0.001.

To confirm the metabolite profiling data, we determined the mRNA expression levels of hypothalamic genes involved in multiple metabolic processes in fasted and control mice using qPCR. As reactive gliosis occurred in the hypothalami of the calorie-restricted model [11], we confirmed the elevated mRNA expression of *glial fibrillary acidic protein* (*Gfap*), the molecular marker for astrocytes in fasted hypothalami (Figure 3A). Short-term food restriction resulted in an increase in the mRNA levels of orexigenic AgRP and a decrease in those of anorexigenic POMC (Figure 3B,C). We subsequently evaluated the membrane transporters of glucose and monocarboxylates in fasted hypothalami to clarify whether the shuttling of the fuel sources responds to food deprivation. We found a significant increase in the *glucose transporter 1* (*Glut1*) mRNA level in fasted hypothalamus, whereas no difference in *glucose transporter 3* (*Glut3*) mRNA level was observed (Figure 3D,E). In addition, we observed that the expression of the *fatty acid transport protein* (*Fatp*) and the *monocarboxylate transporter 1* (*Mct1*), a member of the solute carrier (SLC) family of proteins, genes was not altered in the hypothalami of fasted mice (Figure 3F,G). In support of the metabolite analysis showing increased levels of β-hydroxybutyric acid, we observed that fasted mice had increased mRNA levels of genes involved in the biosynthesis of ketone bodies, including *hydroxymethylglutaryl-CoA lyase* (*Hmgcl*) and *hydroxymethylglutaryl-CoA synthase* (*Hmgcs*) (Figure 3H,I). However, no significant difference of the hypothalamic *lactate dehydrogenase* (*Ldh*) mRNA level was observed in fasted mice, compared to the fed control mice (Figure 3J). These results indicate that the limited systemic availability of energy sources leads to altered patterns of nutrient utilization in the hypothalamus.

### 2.4. Hypothalamic Astrocytes Showed an Altered Expression of Genes Related to Cellular Metabolic Processes in Response to Short-Term Food Deprivation

**Figure 4:**
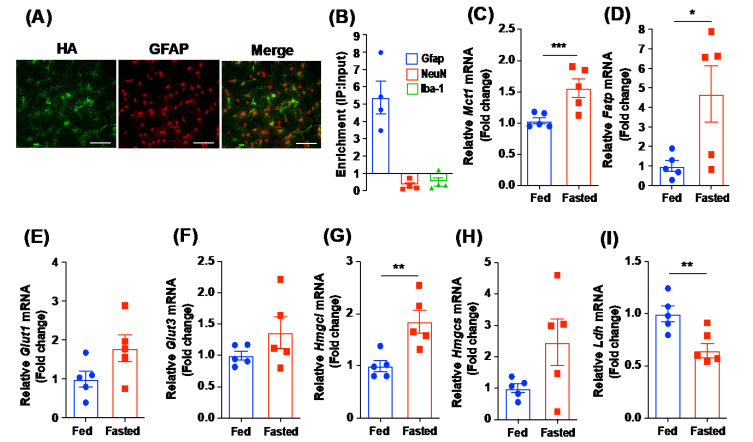
Altered mRNA levels of genes involved in metabolic processes in purified mRNA of hypothalamic astrocyte in response to short-term food deprivation. Astrocyte-specific expression patterns of mRNA were measured in both control and fasted mice using qPCR and Ribo-tag experiments. (**A**) Representative images showing the co-localization of HA and GFAP immunosignals in the hypothalamic arcuate nucleus. (**B**) Purification of astrocytic mRNA was confirmed by evaluating the enrichment of *Gfap*, *NeuN*, and *Iba1* expressions. Fasted mice showed a significant elevation in the levels of astrocytic mRNA involved in metabolic processes, including (**C**) *Mct1*, (**D**) *Fatp*, and (**G**) *Hmgcl*, and a tendency toward increased mRNA levels of (**E**) *Glut1*, (**F**) *Glut3*, and (**H**) *Hmgcs* in the hypothalamic astrocytes. (**I**) A significant decrease in astrocytic *Ldh* mRNA was observed in the hypothalamus of the fasted mice. Data are presented as the mean ± SEM. *n* = 5 mice per group. * *p* < 0.05, ** *p* < 0.01, *** *p* < 0.001. Scale bar = 50 μm.

To confirm whether hypothalamic astrocytes may show a similar trend to our metabolomics data, we analyzed the expression patterns of multiple genes involved in the transportation and production of nutrients in hypothalamic astrocytes using the Ribo-Tag technique with Gfap-Cre;Rpl22^HA^ mice that expressed the HA-tagged ribosomal protein Rpl22 in astrocytes. Astrocyte-specific Cre recombination was confirmed by identifying the immunosignals of the HA protein in the Gfap-positive hypothalamic astrocytes using immunohistochemistry (Figure 4A). We validated the purification of astrocytic mRNA by identifying the predominant expression of *Gfap* mRNA, a molecular marker for astrocytes, compared to the expression levels of *ionized calcium-binding adapter molecule 1* (*Iba-1*) mRNA, a molecular marker for microglia, and *neuronal nuclear protein* (*NeuN*) mRNA, a marker for neurons (Figure 4B). We observed that fasted mice displayed elevated mRNA expression levels of *Mct1* and *Fatp* in hypothalamic astrocytes (Figure 4C,D). *Glut1* and *Glut3* mRNA levels tended to increase in hypothalamic astrocytes in fasted mice (Figure 4E,F). As with the data obtained from metabolomics and gene expression experiments with the total hypothalamus, we observed that fasted mice showed a significant increase in mRNA expression of *Hmgcl* and a tendency toward an increased mRNA expression of *Hmgcs* in hypothalamic astrocytes (Figure 4G,H). Furthermore, we confirmed that fasted mice displayed a significant decrease in *Ldh* mRNA in hypothalamic astrocytes (Figure 4I). Overall, this indicates that hypothalamic astrocytes retained altered cellular metabolic processes directly associated with the data observed in metabolite profiling during short-term food deprivation.

## 3. Discussion

Considerable attention has been paid to identifying the physiological and pathological relevance of metabolic activities in hypothalamic cells in whole-body energy homeostasis. Metabolic coupling between neurons and glial cells in the hypothalamus is an important cellular event that drives appropriate behavioral outputs [5,6]. In this study, hypothalamic metabolites were profiled in response to short-term food deprivation and related molecular data were obtained. Previous studies indicated that the types and patterns of fuel utilization in hypothalamic cells, including appetite-regulating neurons, astrocytes, and microglia, are directly connected to the regulation of energy metabolism [5,12,13]. For instance, altered mitochondrial dynamics occurred in hypothalamic neurons of rodent models of obesity, in which cellular metabolism was genetically modified [4]. We identified that multiple nutrients, which can be used for neuronal excitability and synaptic plasticity, were altered in the fasted hypothalami. γ-aminobutyric acid (GABA) released from AgRP neurons exerts the orexigenic responses via the inhibition of POMC neurons [3,14]. We confirmed that fasted mice showed an increase in GABA content in the hypothalamus. Previous studies showed that alterations in leucine metabolism are coupled to a variety of metabolic processes [15,16]. In agreement with these findings, we confirmed that the levels of hypothalamic leucine were elevated in fasted mice retaining altered metabolic activity, such as hyperphagia and hypoglycemia. Lactate exerts neuronal activity in hypothalamic neurons that controls whole-body energy metabolism, and the shuttling of lactate from tanycytes to POMC neurons is functionally linked to the control of feeding behavior and energy expenditure [17,18]. These previous findings are consistent with our metabolomics results revealing a reduced level of lactate in the hypothalamus of fasted mice. Interestingly, short-term food deprivation did not alter the level of pyruvate in the hypothalamus, despite the decline in lactate biosynthesis. These findings may be associated with the metabolic shift to enhance the tricarboxylic acid cycle during limited nutrient availability. Ketone bodies are known to be produced by the liver and used as an alternative energy source for normal brain function when glucose availability is reduced [19]. We focused on the production of ketone bodies and expression patterns of genes involved in ketogenesis in fasted hypothalami and found, in addition to the reduced lactate content in the hypothalami, a dramatic elevation of levels of β-hydroxybutyric acid in the hypothalami and serum of fasted mice. In line with these findings, previous studies have reported that treatment with synthetic β-hydroxybutyric acid promotes the feeding behavior in mice [20] and enhances the expression of AgRP, an orexigenic peptide [21]. These observations suggest that opposite levels of lactate and ketone bodies could be extracellular messengers, used to determine the ratio of orexigenic and anorexigenic tones. Elevated circulating levels of free fatty acids have been confirmed during restricted carbohydrate availability and the liver utilizes these lipid species to produce ketone bodies, which can serve as an alternative fuel source, especially for the brain and heart [19,22]. Intriguingly, no alteration of fatty acid levels was found in the hypothalamic tissue of fasted mice. These suggest that hypothalamic cells may rely on extracellular fatty acids, rather than cell-autonomous fatty acids to produce ketone bodies. Changes in the physical transportation and biochemical processes of fuel sources in the vessel–astrocyte-neurons axis of the hypothalamus are important in the hypothalamic circuit regulating whole-body energy metabolism. Considerable effort has been made to unveil the functional role of hypothalamic astrocytes in the hypothalamic control of energy metabolism [6,7,10,11,23,24]. For instance, the modification of glucose and fatty acid availability in hypothalamic astrocytes altered the susceptibility to obesity development in association with altered activities of hunger- and satiety-regulating neurons in the hypothalamic arcuate nucleus [6,7]. In the present study, we confirmed the patterns of metabolite content and gene expression in hypothalamic tissues by analyzing the astrocyte-specific expression patterns of genes involved in the shuttling and synthesis of nutrients, such as glucose, fatty acids, and monocarboxylates. In support of the metabolite data, we identified the elevated expression of genes linked to the synthesis of ketone bodies and transportation of monocarboxylates in purified astrocytic mRNA. We recently reported that enhanced lipid utilization occurred in the hypothalamus of mice, retaining the negative energy balance. Intriguingly, an increase in multiple fatty acids and a specific ketone body was observed in the hypothalami of the mice, revealing sickness responses, including severe anorexia [12]. Taken together, we suggest that enhanced ketogenesis and lipid utilization during the development of negative and positive energy balances could be physiological signals to operate the hypothalamic neuronal circuit, as well as the biochemical processes that utilize alternative fuel sources. Further studies, including molecular profiling and pathway analysis, are required to better understand the dynamic changes of metabolic processes in the hypothalamus in response to altered nutrient availability

Collectively, the current findings provide a better understanding of the underlying mechanism of appetite regulation and offer new strategies to be considered in treating patients with eating disorders observed in severe human diseases and obesity.

## 4. Materials and Methods

### 4.1. Animals

Eight-week-old male C57BL/6 mice (Dae Han Bio Link, Seoul, Korea) were housed in a 12 h light-dark cycle at 25 °C and 55 ± 5% humidity. The mice were allowed access to a normal diet and tap water ad libitum. For the food deprivation experiments, food was withdrawn for 18 h starting at 5:00 p.m. Mice were sacrificed by decapitation and their serum and hypothalami were quickly harvested for the designed experiments. All animal care and experimental procedures were performed in accordance with a protocol approved by the Institutional Animal Care and Use Committee (IACUC) at the Incheon National University (permission number: INU-2016-001).

### 4.2. Ribo-Tag Analysis

To evaluate astrocyte-specific mRNA expression in the hypothalamus, we utilized the Ribo-Tag translational profiling system [24,25]. To obtain Ribo-tag mice (Gfap-CreER^T2^: Rpl22^HA^), Rpl22^HA^ mice (Stock No. 011029, Jackson Laboratory, Bar Harbor, ME, USA) were crossbred with glial fibrillary acidic protein (Gfap)-CreERT2 mice (Stock No. 012849), which specifically express Cre recombinase in astrocytes. Because the Gfap-CreER^T2^ mice expressed Cre recombinase under control of the GFAP promoter induced by tamoxifen, 8-week-old Gfap-CreER^T2^: Rpl22^HA^ mice were administered daily intraperitoneal injections of tamoxifen for 3 days (100 mg/kg, T5648, Sigma-Aldrich, St. Louis, MO, USA) dissolved in corn oil (C8267, Sigma-Aldrich). RNA was isolated using the Ribo-Tag system, as previously described [24,25]. Briefly, the hypothalamus was harvested and homogenized before RNA extraction. RNA was extracted from 10% of the cleared lysate and used as an input control. The remaining lysate was incubated with mouse anti-HA antibody for 4 h at 4 °C, followed by the addition of protein-G agarose beads (LGP-1018B, Lugen, Gyeonggi-Do, South Korea) and overnight incubation at 4 °C. The beads were washed three times in a high-salt solution. The bound ribosomes and RNA were separated from the beads by 30 s of vortexing. Total RNA was extracted using a Qiagen RNeasy Micro Kit (74034, Hilden, Germany), according to the manufacturer’s instructions, and quantified with a NanoDrop Lite (Thermo Fisher Scientific, Waltham, MA, USA). To evaluate the levels of ribosome-associated mRNA in the astrocytes, we synthesized cDNA using a high-capacity cDNA reverse transcription kit (Applied Biosystems, Foster City, CA, USA) and performed quantitative real-time PCR.

### 4.3. Quantitative Real-Time Reverse Transcription-Polymerase Chain Reaction

Total RNA was extracted from the hypothalamus, according to the Tri-Reagent protocol, and cDNA was then synthesized from total RNA and ribosome-associated mRNA using a high-capacity cDNA reverse transcription kit (Applied Biosystems, Foster City, CA, USA). Amplification of the cDNA was detected using the SYBR Green Real-Time PCR Master Mix (Toyobo Co., Ltd., Osaka, Japan) in a Bio-Rad CFX 96 Real-Time Detection System (Bio-Rad Laboratories, Hercules, CA, USA). The results were analyzed by the CFX Manager software and normalized to the levels of *β-actin* housekeeping genes. All reactions were performed under the following conditions: initial denaturation at 95 °C for 3 min, followed by 40 cycles of 94 °C for 15 s, 60 °C for 20 s, and 72 °C for 40 s. The primers used were as follows: *Gfap*, F-TCAATGACCGCTTTGCTAGC and R-ACTCGTGCAGCCTTACACAG; *Iba-1*, F-TCTGCCGTCCAAACTTGAAG and R-TCTAGGTGG GTCTTGGGAAC; *NeuN*, F-ATGGTGCTGAGATTTATGGAGG and R-CGATGGTGTGATGGTAAGGATC; *Agrp*, F-TGTGTAAGGCTGCACGAGTC and R-GGCAGTAGCAAAAGGCATTG; *Pomc*, F-TCCTACTCCATGGAGCACTTC and R-TCCTACTCCATGGAGCACTTC; *Glut1*, F-CTTCATTGTGGGCATGTGCTTC and R-AGGTTCGGCCTTTGGTCTCAG; *Glut3*, F-TCTGTTGGTGGCATGATTGG and R-ATGATGGCCAGCAAGTTGAC; *Ldh*, F-AGCCCTGACTGCACCATCATC and R-CGGAATCGAGCAGAATCCAGA; *Mct1*, F-AATGATCGCTGGTGGTTGTC and R-TTGAAAGCAAGCCCAAGACC; *Fatp*, F-GCAGCATTGCCAACATGGAC and R-GTGTCCTCATTGACCTTGACCAGA; *Hmgcs*, F-TTTGATGCAGCTGTTTGAGG and R-CCACCTGTAGGTCTGGCATT; *Hmgcl*, F-CCAGCTTTGTTTCTCCCAAG and R-TCAGACACAGCACCGAAGAC; *β-actin*, F-TGGAATCCTGTGGCATCCATGAAAC and R-TAAAACGCAGCTCAGTAACAGTAACAGTCCG.

### 4.4. Immunohistochemistry

The mice were transcardially perfused with 0.9% saline (*w*/*v*), followed by perfusion with fresh fixative composed of 4% paraformaldehyde in a phosphate buffer (PB, 0.1 M, pH 7.4). The brains were collected and post-fixed overnight before coronal sections (50 μm thickness) were prepared using a vibratome (5100 mz Campden Instruments, Leicestershire, UK). After washing in PB several times, the sections were pre-incubated with 0.3% Triton X-100 (Sigma-Aldrich) in PB for 30 min at room temperature (RT) and incubated overnight with rabbit anti-GFAP antibody (1:1000 dilution, ab7260, Abcam, Cambridge, UK) or mouse anti-HA antibody (1:1000; MMS-101R, BioLegend, San Diego, CA, USA) at RT. Immunofluorescence was performed with secondary antibodies (Alexa Fluor 488-labeled anti-mouse antibody, 1:500; A11001, Invitrogen, Carlsbad, CA, USA or Alexa Fluor 594-labeled anti-rabbit antibody, 1:500; A21209, Invitrogen) for 2 h at RT. The sections were mounted onto glass slides and covered with coverslips using a drop of mounting medium (Dako North America, Carpinteria, CA, USA). The coverslips were sealed with nail polish to prevent the desiccation and movement of the samples under the microscope. Images were obtained using fluorescence microscopy (Axioplan2 Imaging, Carl Zeiss Microimaging, Oberkochen, Germany) and then subjected to analyses.

### 4.5. Sample Extraction and GC-MS Analysis

The extraction and analysis procedures from the serum and hypothalamus were described in a previous report [12,26]. Briefly, the serum (0.1 mL) was extracted with 0.3 mL of 3:1 (*v*/*v*) methanol:chloroform and 0.03 mL of internal standard (IS, 2-chloro-L-phenylalanine, 300 μg/mL). The hypothalamus was extracted after homogenizing it 3 times for 20 s with 250–300 mg glass beads of 425–600 μm, 1 mL of 50% methanol (*v*/*v*), and 0.03 mL of IS using a bead beater (Mini Beadbeater 96, BioSpec Products, Bartlesville, OK, USA). The sample extracts were sonicated for 10 min and centrifuged at 13,000× *g* for 15 min. The supernatant liquid separated into a clean tube was completely dried using a vacuum concentrator (VS-802F, visionbionex, Gyeonggi-do, Korea) and a freeze dryer (MCFD8512, IlShinBioBase, Gyeonggi-do, Korea) for about 4 h and 16 h, respectively. The freeze-dried sample was derivatized in 80 μL of methoxyamine hydrochloride (20 mg/mL) at 37 °C for 90 min, and then mixed with 80 μL of N,O-Bis(trimethylsilyl) trifluoroacetamide at 60 °C for 60 min. The qualitative and quantitative analyses of GC-MS data were performed using a previously described method [12].

### 4.6. Statistical Analysis

The normalization (unit variance scaling) of the identified GC-MS data was performed for partial least square discriminant analysis (PLS-DA). PLS-DA was conducted using the soft independent modeling of class analogy (SIMCA) package 14.1 software (Umetrics, Umeå, Sweden). Pearson’s correlation analysis was conducted using SAS software 9.4 (SAS Institute Inc., Cary, NC, USA). Hierarchical clustering analysis was performed using Multi-Experiment Viewer version 4.9. Data were statistically analyzed using analysis of variance (ANOVA) followed by Student’s t-test using Prism GraphPad. A *p*-value ≤ 0.05 was considered statistically significant. The values are represented as the means ± standard error of the mean (SEM).

## Data Availability

All data reported in the manuscript.

## Supplementary Materials

The following supporting information can be downloaded at: https://www.mdpi.com/article/10.3390/metabo12050407/s1, Figure S1: VIP plots of the OPLS-DA models obtained from 44 sera and 45 hypothalamic metabolites from fasting and control mice; Figure S2: Correlation matrix of the 45 metabolites identified from the hypothalamus of mice.
