# Supplementary material for: Metabolic Profiling of the Hypothalamus of Mice during Short-Term Food Deprivation
Source: Metabolites. 2022 Apr 29;12(5):407. doi: 10.3390/metabo12050407 (PMC9144291; doi:10.3390/metabo12050407)
Supplement: Supplementary file 1 [file metabolites-12-00407-s001.zip › metabolites-1654497-supplementary.pdf]

# Metabolic profiling of the hypothalamus of mice during short-term food deprivation

**Ye Jin Kim [1,#], Dasol Kang [2,#], Hye Rim Yang [1], Byong Seo Park [1], Thai Hien Tu [1], Bora Jeong [2], Byung Ju Lee [2], Jae Kwang Kim [1,*] and Jae Geun Kim [1,*]**

[1] Division of Life Sciences, College of Life Sciences and Bioengineering, Incheon National University, Incheon 22012, South Korea; 201721047@inu.ac.kr (Y.J.K.); hr.yang0414@inu.ac.kr (H.R.Y.); 2021s135@inu.ac.kr (B.S.P.); thaihientu@gmail.com (T.H.T.)

[2] Department of Biological Science, University of Ulsan, Ulsan 44610, South Korea; lainef7@nate.com (D.S.); boraring@naver.com (B.J.); bjlee@ulsan.ac.kr (B.J.L.)

* Correspondence: jgkim@inu.ac.kr (J.G.K.); kjkpj@inu.ac.kr (J.K.K.); Tel.: +82-32-835-8256 (J.G.K.); Tel.: +82-32-835-8241 (J.K.K.)

[#] These authors contributed equally to this work.

## Supplementary File

**Figure S1.** VIP plots of the OPLS-DA models obtained from 44 sera (A) and 45 hypothalamic (B) metabolites from fasted and control mice.

**Figure S2.** Correlation matrix of the 45 metabolites identified from the hypothalamus of fasted and control mice.

**(A) Serum**

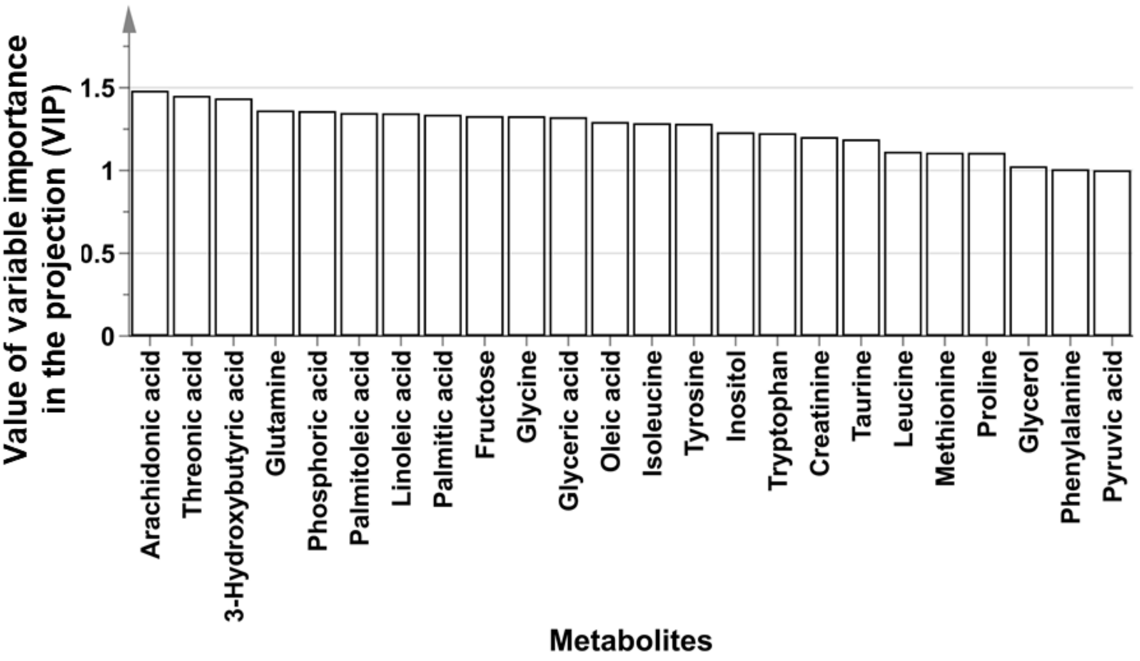


**(B) Hypothalamus**

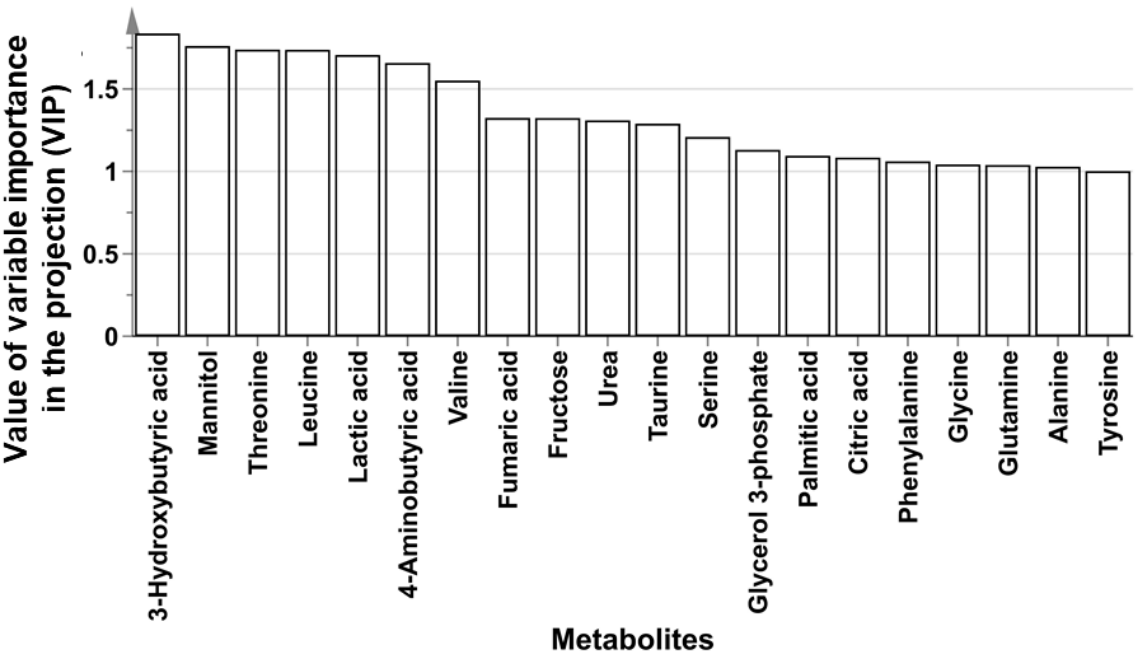


**Figure S1.** VIP plots of the OPLS-DA models obtained from 44 sera (A) and 45 hypothalamic (B) metabolites from fasted and control mice. Metabolites with VIP values greater than 1.0 are displayed on the VIP plot.

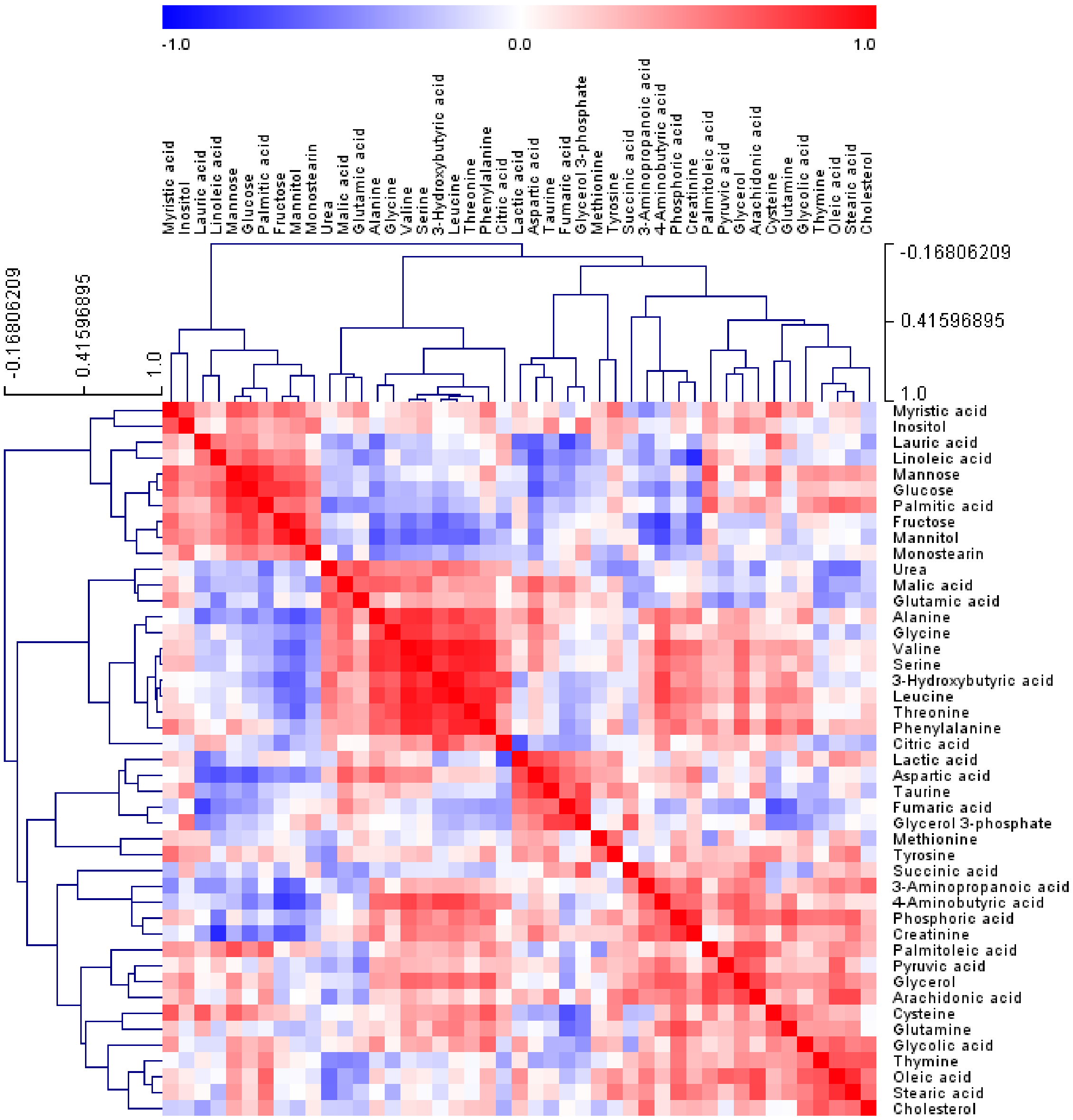


**Figure S2**. Correlation matrix of the 45 metabolites identified from the hypothalamus of fasted and control mice. Each square represents the Pearson's correlation coefficient by the intensity of the blue or red color.
